# Filling the Knowledge Gap Regarding Microbial Occupational Exposure Assessment in Waste Water Treatment Plants: A Scoping Review

**DOI:** 10.3390/microorganisms12061144

**Published:** 2024-06-04

**Authors:** Bruna Riesenberger, Margarida Rodriguez, Liliana Marques, Renata Cervantes, Bianca Gomes, Marta Dias, Pedro Pena, Edna Ribeiro, Carla Viegas

**Affiliations:** 1 H&TRC—Health & Technology Research Center, ESTeSL—Escola Superior de Tecnologia e Saúde, Instituto Politécnico de Lisboa, 1990-096 Lisbon, Portugal; 2 NOVA National School of Public Health, Public Health Research Centre, Comprehensive Health Research Center, CHRC, REAL, CCAL, NOVA University Lisbon, 1099-085 Lisbon, Portugal

**Keywords:** wastewater treatment plants, sampling methods, assays, microbial contamination assessment, bacteria, fungi

## Abstract

Background: Wastewater treatment plants (WWTPs) are crucial in the scope of European Commission circular economy implementation. However, bioaerosol production may be a hazard for occupational and public health. A scoping review regarding microbial contamination exposure assessment in WWTPs was performed. Methods: This study was performed through PRISMA methodology in PubMed, Scopus and Web of Science. Results: 28 papers were selected for data extraction. The WWTPs’ most common sampled sites are the aeration tank (42.86%), sludge dewatering basin (21.43%) and grit chamber. Air sampling is the preferred sampling technique and culture-based methods were the most frequently employed assays. *Staphylococcus* sp. (21.43%), *Bacillus* sp. (7.14%), *Clostridium* sp. (3.57%), *Escherichia* sp. (7.14%) and *Legionella* sp. (3.57%) were the most isolated bacteria and *Aspergillus* sp. (17.86%), *Cladosporium* sp. (10.71%) and *Alternaria* sp. (10.71%) dominated the fungal presence. Conclusions: This study allowed the identification of the following needs: (a) common protocol from the field (sampling campaign) to the lab (assays to employ); (b) standardized contextual information to be retrieved allowing a proper risk control and management; (c) the selection of the most suitable microbial targets to serve as indicators of harmful microbial exposure. Filling these gaps with further studies will help to provide robust science to policy makers and stakeholders.

## 1. Introduction

The European Commission (EC) strongly recommends circular economy implementation aiming at a zero-waste strategy, by instigating water innovations technologies for water reuse and recycling [1]. In this scope, wastewater treatment plants (WWTPs) are designed to maximize energy and water recovery, becoming of pivotal importance for the achievement of the EC’s goals [2]. 

On WWTPs, the wastewater of domestic, hospital and industrial uses undergoes preliminary, primary, secondary, and in some cases, tertiary biological treatments [3,4]. During these treatments, bioaerosol formation is higher throughout discharging, mixing and aerating processes, as well as during the spraying of sewage [3,4,5,6]. The bioaerosols contain microorganisms, such as fungi, viruses, bacteria, and their metabolites, including endotoxins and mycotoxins, which may be potentially pathogenic to humans. Infection can occur through ingestion, dermal contact, or inhalation, and it is highly possible that due to prolonged exposure, a decline in the health status of WWTPs workers may be observed [5,6,7]. In fact, several negative health outcomes associated with bioaerosol occupational exposure have been reported, including respiratory and gastrointestinal effects or allergies [4,6]. In addition, WWTPs are recognized as key emission sources for the discharge of antimicrobial-resistant (AMR) bacteria and antibacterial resistance genes (ARGs) [8].

Although it is crucial to assess occupational exposure to bioaerosols in WWTPs, there is a lack of consensus regarding sampling approaches and analyses that should be performed, as well as the targets that can be used as surrogates to identify harmful microbial contamination, which is a common problem in settings where (micro)biologic agents need to be assessed. However, suggestions regarding the procedures to be employed from the field to lab have already been described in different occupational environments [9,10,11]. Thus, this study aims to perform a scoping review to provide a broad overview of the state-of-the-art methods (sampling and analyses) applied to perform microbial contamination assessments in WWTPs, as well as to identify the most suitable targets to be used as indicators of hazardous microbial contamination.

## 2. Materials and Methods

### 2.1. Search Strategy, Inclusion and Exclusion Criteria 

This study adopted the PRISMA methodology and the Preferred Reporting Items for Systematic Review (PRISMA) checklist [12] (was completed (Supplementary Materials Table S1).

This study reports available data published between 1 January 2010 and 8 November 2023. The search aimed at selecting studies on microbiologic agents’ assessment in WWTP and included the terms “Waste Water Treatment Plants”, “bacteria”, “fungi”, “viruses”, “exposure” and “sampling”, with English as the chosen language. The databases chosen were PubMed, Scopus and Web of Science (WoS). Articles that did not meet the inclusion criteria were not subjected to additional review (Table 1). 

### 2.2. Studies Selection and Information to Be Retrieved from the Papers

The articles were selected by using the Rayyan—Intelligent systematic review tool, a free online tool that significantly accelerates the process of screening and choosing papers for academics working on systematic reviews. Article selection followed three rounds: 1st: All titles were screened to identify and remove duplicated papers or those unrelated to the topic. The selected papers were uploaded to Rayyan for additional examination; 2nd: screening of all the abstracts; 3rd: Selected papers were reviewed considering the inclusion and exclusion criteria. Possible differences in the study’s selection were discussed by three investigators (BR, MR and LM), and eventually decided by the remaining investigators (BG, MD, PP, RC). Data extraction was then performed by two investigators (BR, and LM), while another (MR) examined the results. The data that follows were manually extracted: Database, Title, Country, Type of WWTP, Sampling Strategies and Methods, Assays applied, Main Findings, and References. 

### 2.3. Quality Assessment

The assessment of the risk of bias was performed by 4 investigators (BR, MR, LM, and CV). Within each research article, an evaluation of the risk of bias was performed across two parameters divided as key criteria (“Sampling methods” and “Assays”). Each parameter’s risk of bias was rated as “low” “medium” “high”, or “not applicable”. The studies for which all the key criteria and most of the other criteria were characterized as “high” were removed.

## 3. Results

**Figure 1:**
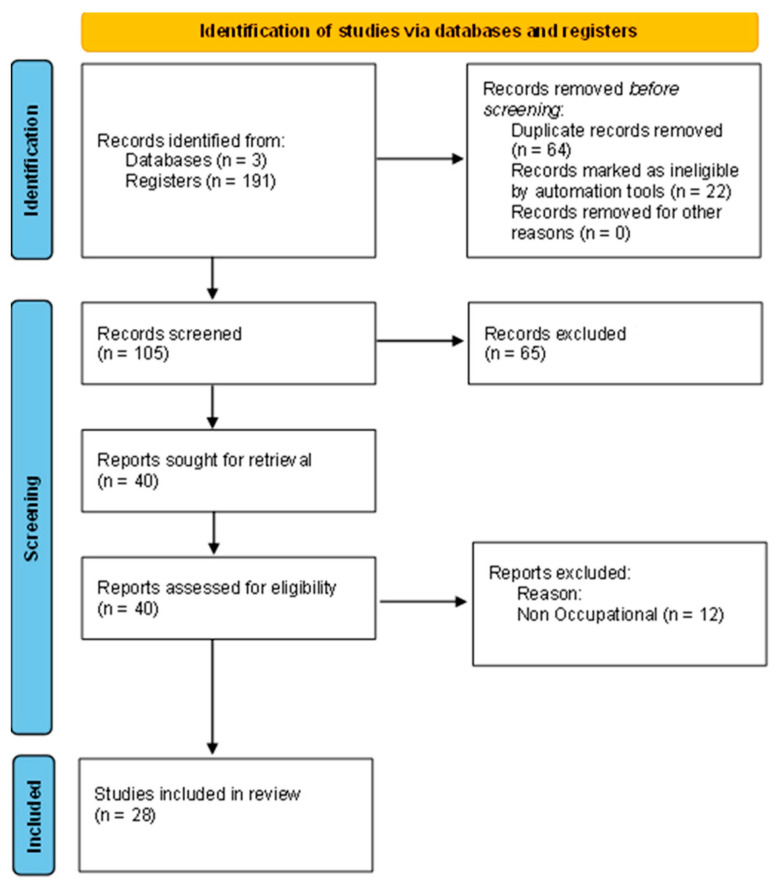
PRISMA methodology of selection of papers [12].

The workflow illustrated in Figure 1 was used for selecting studies. Initially, 191 studies were found in the database search, from which 105 abstracts were analyzed, and 40 complete texts were deemed eligible for further examination. A total of 12 papers were rejected for not satisfying the inclusion and exclusion criteria, mostly because they did not have any information regarding microbial occupational exposure in WWTPs. Overall, the selection process yielded 28 studies on microbiologic contamination occupational exposure assessment.

### Extracted Data

After the selection of the 28 studies on microbiologic contamination occupational exposure assessment, the relevant data were extracted; the key findings are summarized in Table 2. 

Among the 28 chosen studies, 9 were conducted in Europe (3 in Portugal [13,14,15], 2 in Poland [16,17], 2 in Denmark [18,19], 1 in Switzerland [20], and 1 in Austria [21]), 9 in Asia (specifically in China [4,5,22,23,24,25,26,27,28]), 6 in the Middle East (Iran [6,29,30,31,32,33]), and 4 in North America (3 in the USA [34,35,36] and 1 in Canada [37]).

Among the chosen studies, 12 (42.86%) were conducted within Municipal WWTP [4,6,15,17,22,24,25,28,29,30,31,37]. However, information regarding the type of WWTP was not explicit in 16 studies (57.14%) [5,13,14,16,18,19,20,21,23,26,27,32,33,34,35,36].

The most common sampled sites were the aeration tank (42.86%) [6,16,18,19,22,24,26,27,28,29,30,33], sludge dewatering basin (21.43%) [6,13,27,28,29,30] and grit chamber (17.86%) [6,19,27,29,30,33]. Some authors choose to perform the sampling at 1.5 m up on the aeration tanks (7.14%) [18,22]. Only one study (3.57%) [23] focused on sampling at different distances from the rotation brushes.

**Table 2:** Data extracted from the chosen papers.

Title	Type of WWTP	Microbe Assessed	Sampling Methods	Season of Sampling	Sampling Sites and Number of Samples	Assays	Main Findings	Reference
Exposure to Airborne Noroviruses and Other Bioaerosol Components at a Wastewater Treatment Plant in Denmark	no data	Noroviruses (NoVs), Adenoviruses, Endotoxin, Bacteria and Molds	Air samples: Active methods—Filtration (Personal Dust Sampling-Inhable GSP samplers with teflon filters or polycarbonate filters, average sampling period 242 min), Stationary measurements of “total dust”	no data	Personal dust sampling was carried out on 16 workers, on different wastewater processes; stationary sampling was carried out in the aeration basin at 1.5 m above the ground level	Culture-based methods (DG18 agar for cultivable moulds, nutrient agar for cultivable bacteria)	NoVs and endotoxin were detected at concentrations that could pose an occupational health risk. Positive correlations between exposure to endotoxin, bacteria, moulds and NoVs were found and indicate that the exposure to bioaerosols may be related to work tasks.	[18]
ADMS simulation and influencing factors of bioaerosol diffusion from BRT under different aeration modes in six wastewater treatment plants	Municipal WWTP	Bacteria and Intestinal Bacteria	Air samples: Active methods—Impaction (Andersen six-stage cascade impactor, flow rate = 28.3 L/min; TH-150 medium flow sampler)	Seasonal (spring)	1.5 m above aeration tanks of 6 Municipal Wastewater Treatment Plant (MWWT), 6 samples were taken at each sampling site, (n = 36)	Culture-based methods (LB medium for bacteria, and for intestinal bacteria, MAC); Ion chromatography and Illumina MiSeq high-throughtput sequencing	The concentrations of bacteria and, specifically, intestinal bacteria in the bioaerosols ranged from 389 CFU/m^3^ to 1536 CFU/m^3^ and 30 CFU/m^3^ to 152 CFU/m^3^, respectively, and the proportion of the intestinal bacteria was 8.85%. The proportion of intestinal bacteria (75.79%) produced via surface aeration by Biological Reaction Tanks (BRT) attached to large-sized bioaerosol particles was higher than that of a BRT undergoing the bottom aeration process (37.28%). The main microorganisms found in the bioaerosols included Moraxellaceae, Escherichia–Shigella, Psychrobacter, and Cyanobacteria.	[22]
Spatio-temporal variations in airborne bacteria from the municipal wastewater treatment plant: a case study in Ahvaz, Iran	Municipal WWTP	Airborne Bacteria	Air Samples: Passive methods (microbiological sampling index of microbial air contamination (1/1/1 standard))	Seasonal (warm and cold)	Grit chamber (GCh), primary sludge dewatering basin (PSDB) and at the aeration tank (AT); (n = 180)	Culture-based methods (Trwith nystatin (250 mg/L) to inhibit fungal growth); PCR-RFLP	The dominant bacterial genus included *Bacillus pumilus* (26.7%), *Staphylococcus arlettae* (23.2%), *Kocuria turfanensis* (13.6%), and *Alicycliphilus* (9.2%), and they increased with high temperatures and wind speed, and decreased with high humidity.	[6]
Emission level, particle size and exposure risks of airborne bacteria from theoxidation ditch for seven months observation	WWTP with orbal oxidation ditch process	Airbone Bacteria	Air samples: Active—Impaction (Andersen six-stage cascade impactor, flow rate = 28.3 L/min); Material collection (raw water in the oxidation ditch)	Seasonal (spring and summer)	ConS: Control site was set 300 m upwind from the oxidation ditch; AWS (above water surface): above water surface; AWS-0.5: above water surface 1 m; AWS-3: above water surface 3 m; ARB (above rotating brushes)-25: after the rotating brushes 25 m; ARB-55: after the rotating brushes 55 m; ARB-210: after the rotating brushes 210 m; (n = 168)	Culture-based methods (with nutrient agar for mesophilic bacteria) for air samples; Gradient gel electrophoresis for 16S rDNA; PCR	Spatial and seasonal variations in the concentrations of airborne bacteria emissions were detected. The highest concentration was observed near the rotor disc aerators (RDAs) (835 ± 91 CFU/m^3^ to 8916 ± 155 CFU/m^3^) during each sampling process, with the concentration decreasing by 76.70% and 79.91% as sampling distance and height increased, respectively. Most of the airborne bacteria were coarse particles that exceeded 4.7 μm in size. The dominant bacteria were *Bacillus* sp., *Lysinibacillus* sp., and *Sphingomonas* sp.	[23]
Aerosol partitioning potential of bacteria presenting antimicrobial resistance from different stages of a small decentralized septic treatment system	On-site/decentralized WWTP	Antibiotic-Resistant Bacteria (ARB)	Air samples: Active method—Impinger (Wetted wall cyclone collectors (WWC)); Material collection (stainless steel porTable 600 mL water dipper (Grainger Industrial Supply))	Seasonal (summer and winter)	Aerosol and water samples were collected at the four tanks; 600 mL of water and 1500 L of air at each tank	Kirby–Bauer testing for antibiotic resistance, quantitative Polymerase Chain Reaction (qPCR); 16SrRNA-based Illumina sequencing	As expected, the higher concentration of bacteria was found when the lids were open; in the summer, *Legionella* was found in the water tanks 1 and 3, and in the water tank 1 *Pseudomonas* was present; in the winter, *Legionella* was also present in the water tank 1; bioaerossol samples showed a higher antimicrobial resistance of 50% (at four of the eight antbiotics tested), and the higher antimicrobial resistance of the water samples was 87.5% (resistance in the 7 of the 8 antibiotics).	[36]
Identification of airborne fungi’s concentrations in indoor and outdoor air of municipal wastewater treatment plant	Municipal WWTP with conventional activated sludge treatment process	Fungi	Air Samples: Passive methods (microbiological sampling index of microbial air contamination (1/1/1 standard))	Seasonal (warm and cold)	Grit chamber tank, primary sludge dewatering basin, aeration tank, upstream and downstream of dominant wind blowing at the site and at the administrative building (n = 240)	Culture-based methods (SDA with chloramphenicol antibiotic (100 mg/L) to inhibit bacterial growth)	The greatest release of fungal aerosols occurred in the cold season while the minimum release occurred in the warm season; the highest concentrations of fungi were observed in the grit chamber unit; *Cladosporium* (39.23%) and *Alternaria* (19.87%) were the airborne fungal genera most common.	[29]
Aspergillus spp. prevalence in different Portuguese occupational environments: What is the real scenario in high load settings?	no data	*Aspergillus* spp.	Air samples: Active methods—Impaction (Millipore air Tester, flow rate = 140 L/min) and Impinger (Coriolis μ air sampler, flow rate = 300 L/min); Passive methods: surface samples (swabs)	1 year longitudinal study	Sampling occured at 2 Watewater Treatment Plant (WWTP); 26 air sample and 15 surface samples	Culture-based methods (MEA); Real Time PCR (RT-PCR)	At both WWTPs were found 33 different species of *Aspergillus* spp. (18 at WWTP1 and 15 at WWTP2), 7 species were only isolated in surfaces (5 in the WWTP1 and 2 at WWTP2), and 12 different Aspergillus sections were identified (6 in both WWTP).	[14]
Wastewater treatment plant workers’ exposure and methods for risk evaluation of their exposure	WWTP with anaerobic–anoxic–oxic process	Airborne Bacteria, Enteric Bacteria, Endotoxins	Air Samples: Active methods—Filtration (personal and stationary GSP samplers with polycarbonate filters or with Teflon filter, flow rate = 3.5 L/min) and Impaction (Andersen six-stage cascade impactor, flow-rate 28.3 L/min)	1 year longitudinal study	Stationary samples were taken in the grid chamber house and in the aeration tank (106 personal GSP samples, 12 stationary GSP samples), and 141.5 L to 843 L of air by ASCI were taken over the year	Matrix-assisted laser desorption/ionization time-of-flight mass spectrometry (MALDI-TOF MS)	A total of 22.36% of the bacteria potentially inhaled by WWTP workers seem to be from the air around the aeration tank and 22.40% from the grid house; *Staphylococcus* (13.2%) and *Aeromonas* (11.7%) were the dominant genera at the aeration tank, while *Acinetobacter* (25.6%) was the dominant in grid house.	[19]
Anaerobic bacteria in wastewater treatment plant	Mechanical–biological WWTP	Anaerobic Bacteria	Air samples: Active method—Impaction (Andersen six-stage cascade impactor, flow-rate = 28.3 L/min); Material collection (sewage and sludge samples were taken directly into 50 mL sterile screwed-off Falcon tubes)	Seasonal (summer and winter)	Bar screens, containers with solids in the screens’ hall, primary settling tank, sewage sludge pumping station, aeration basins incineration plant, sludge-thickening building, and at the background of WWTP (n = 22)	Culture-based methods (Schaedler agar with 5% additive of sheep blood for bacterial growth); PCR (for confirmation of *Clostridium* isolates); Biochemical API 20A test (bioMérieux)	Some of the anaerobic bacteria identified belongs to the risk group 2 (according to the EU Directive 2000/54/EC); *Actinomyces*, *Bifidobacterium*, *Clostridium* and *Propionibacterium* genera were identified in wastewater and in the air.	[16]
Bioaerosols emission characteristics from wastewater treatment aeration tanks and associated health risk exposure assessment during autumn and winter	Municipal WWTP with rotating disc aeration tank, adopted with DE oxidation ditch treatment process, and microporous aeration tank and adopted with Anaerobic–anoxic–oxic process	*Escherichia coli* and *Staphylococcus aureus*	Air samples: Active—Impaction (Andersen six-stage cascade impactor, flow-rate = 28.3 L/min); Material collection (500 mL wastewater samples were taken by a sterility water sampling bottle)	Seasonal (autumn and winter)	The sampling was carried out at 3 WWTPs, and they were located in the middle of the center corridor of the second microporous aeration tank and the first rotating disc aeration tank from north to south	Culture-based methods (for *S. aureus* MYP was used, and MAC for *E. coli*)	*Staphylococcus aureus* was about 2 times higher in winter than in autumn, while *Escherichia coli* in autumn was about 9 times higher than in winter.	[24]
Influence of seasons and sites on bioaerosols in indoor wastewater treatment plants and proposal for air quality indicators	Municipal WWTP with pre-, primary and secondary treatments	Bacteria and Endotoxins	Air samples: Active—Impaction (Andersen six-stage cascade impactor, flow rate = 28.3 L/min) and Filtration (37 mm cassettes (SKC) loaded with binder-free glass fiber filters, flow-rate = 2 L/min)	Seasonal (warm and cold)	Screening, degreasing/grit removal, settling tanks and biofiltration	Culture-based methods (TSA to collect total culturable aerobic bacteria and Gram-negative selective agar (GNSA) for culturable Gram-negative bacteria). In addition to total bacteria (bacteria 16S rDNA), specific qPCR was used to monitor bacteria from human flora: *E. coli*, *Klebsiella pneumonia*, *Pseudomonas aeruginosa*, and fresh water environment: *Aeromonas hydrophila*.	The average concentration of culturable Gram-negative bacteria was approximately 100 CFU/m^3^ for both seasons. Only two WWTPs showed concentrations of culturable Gram-negative bacteria higher than the recommended exposure limit (1000 CFU/m^3^ according to Institut Robert Sauvé en Santé et en Sécurité du Travail (IRSST). Several values were close to the limit.	[37]
Assessment of bioaerosol contamination (bacteria and fungi) in the largest urban wastewater treatment plant in the Middle East	Municipal WWTP with air diffusion by fine bubble diffusers	Airborne Bacteria and Fungi	Air samples: Active method—Impaction (QuickTake 30 sample pump equipped with the Bio Stage single-stage cascade impactor, flow rate = 28.3 L/min)	1 year longitudinal study	Area adjacent to the aeration tank and secondary sedimentation units, near the tricking filter, near the sludge storage tank and sludge dewatering unit, adjacent the screening, grit chamber, and primary sedimentation unit and outside of the WWTP were the locations of the sampling; (n = 240)	Culture-based methods (TSA for airborne bacteria growth, and SDA for fungal growth)	Maximum bacterial concentration was found in the aeration tank in the summer, and the minimum was in the sludge dewatering unit during the winter; maximum and minimum fungal concentrations were in primary treatment and sludge dewatering unit in winter and summer, respectively. *Micrococcus* spp. and *Staphylococcus* spp. had the highest emission of bacteria in the winter and summer, respectively. *Cladosporium* spp., Penicillium spp., *Aspergillus* spp. and *Alternaria* spp. were the dominant fungi.	[30]
Characterization of the airborne bacteria community at different distances from the rotating brushes in a wastewater treatment plant by 16S rRNA gene clone libraries	Municipal WWTP with orbal oxidation ditch treatment process	Airborne Bacteria	Air samples: Active methods—Impaction (six-stage Impacting Airborne Microorganism Sampler—FA-1, 28.3 L/min) and Impinger (SKC BioSampler, flow rate = 12.5 L/min)	no data	Aerosol samples were collected at different distances from the rotating brushes in the oxidation ditch; 1.5 L for each sample	Culture-based methods; PCR; Sequencing	The majority of bacteria in the bioaerosols were *Proteobacteria* and *Bacteroidetes* around the oxidation ditch. The study concluded that the rotating brush aeration was the main source of bioaerosols in the oxidation ditch.	[25]
Genomic insight into transmission mechanisms of carbapenem-producing Citrobacter spp. isolates between the WWTP and connecting rivers	WWTP with anaerobic–anoxic–oxic treatment process	Carbapenem-Resistant *Citrobacter* spp. (CRCS)	Material collection (wastewater and sludge mixtures samples with a total volume of 1 L)	Seasonal (spring, summer, autumn and winter)	Water inlet, anaerobic tank, aerobic tank, sludge thickening tank, activated sludge tank, mud cake storage area, and water outlet; In total, 136 river water and 51 river sediment samples were collected and 189 samples were gathered from the WWTP.	Culture-based methods; PCR; 16s RNA sequencing; MALDI-TOF MS	In total, 14 CRCS were detected in 376 environmental samples, including those from the inlet (n = 7), anaerobic tank (n = 2), and rivers (n = 5). *Citrobacter braakii* (n = 6) was the dominant subtype among 14 CRCS isolates, followed by *Citrobacter freundii* (n = 5), *Citrobacter sedlakii* (n = 2), and *Citrobacter werkmanii* (n = 1). All CRCS showed resistance to the studied antibiotics.	[26]
Aspergillus flavus contamination in two Portuguese wastewater treatment plants	WWTP with primary, secondary and tertiary treatment processes	*Aspergillus* spp.	Air samples: Active methods—Impaction (Millipore, flow rate = 140 L/min) and Impinger (Coriolis µ air sampler, flow rate = 300 L/min); Passive methods: surface samples (swabs)	Seasonal (winter)	Ten sampling locations were established at the two WWTP for assessing indoor air contamination: lift station, flotation sludge, sludge dewatering, screening, co-generation, aerobic digestion (secondary treatment), canteen, operation room, grit removal, and administration room. An outdoor reference sampling was also included; air samples: 26 indoor and 2 outdoor; surface samples: 17 indoor	Culture-based methods (MEA); RT-PCR	In both WWTPs, *Aspergillus versicolor* (38%), *Aspergillus candidus* (29.1%), and *Aspergillus sydowii* (12.7%) were the most common. In the surfaces were *Aspergillus flavus* (47.3%), *Aspergillus fumigatus* (34.4%), and *Aspergillus sydowii* (10.8%). The isolates of *Aspergillus flavus* that were inoculated in coconut agar medium were not identified as toxigenic, and were not detected by RT-PCR.	[13]
Bioaerosol emissions and detection of airbone antibiotic resistance genes from a wastewater treatment plant	Municipal WWTP with activated sludge treatment process	Culturable Bacteria and Fungi; Fluorescent Bioaerosols	Air Samples: Active method—Impaction (Reuter Centrifugal Sampler High Flow, flow-rate = 100 L/min) and Impinger (SKC Biosampler, flow-rate = 12.5 L/min; Particulate matter (Ultraviolet aerodynamic 190 particle sizer (UV-APS)	Seasonal (spring, summer, autumn, and winter)	Sludge thickening basin, biological reaction basin, screen room	Culture-based methods (with TSA and MEA for bacterial and fungal growth, respectively); PCR	Highest concentrations in sludge thickening basin (bacteria: 1697 CFU/m^3^, fungi: 930 CFU/m^3^). Bacterial concentrations met Chinese standards, but fungal levels exceeded World Health Organization (WHO) recommendations in some areas.	[4]
Occupational Exposure to *Staphylococcus aureus* in the Wastewater Treatment Plants Environment	Municipal WWTPs with mechanical, chemical and biological treatments processes	*Staphylococcus aureus*	Air samples: Active methods—Impaction (1-step portable air sampler made by Burkard, flow rate = 20 L/min) and Filtration (GilAir-5 pump and an open-faced aerosol sampler with a gelatin filter of a 37 mm in diameter and 3 µm pores at a flow rate of 3 L/min); Material collection (raw wastewater discharged into the wwtp and treated wastewater)	Seasonal (spring and summer)	The study was conducted in 16 WWTPs in Poland, representing different treatment technologies; a total of 286 samples were collected, including 253 air samples and 33 wastewater samples	Culture-based methods (chromogenic substrate CHROMID^®^ S. aureus Elite agar); MALDI-TOF, and an automatic method for antibiotic resistence na alysis (WalkAway system)	The study identified *Staphylococcus aureus*, including antibiotic-resistant strains, in wastewater and air samples from WWTP. Workers engaged in mechanical treatment faced the highest health risk.	[17]
COVID-19 infection risk from exposure to aerosols of wastewater treatment plants	Municipal with activated sludge treatment process	SARS-CoV-2	Air samples: Active method—Impinger (Portable pumps; flow rate = 7.5–8.5 L/min); Material collection (Grab samples—raw wastewater was colected in 250 mL in sterile glasses)	1 year longitudinal study	Pumping station and activated sludge plants; a total of 24 raw wastewater samples were collected, with 12 samples from each of the two wastewater treatment plants (WWTPs) and 15 air samples.	RT-qPCR	SARS-CoV-2 RNA was found in 37.5% of wastewater samples. Detected in 5 of 12 samples from WWTP A and 4 of 12 samples from WWTP B. The highest concentration was observed at the pumping station.	[31]
Dispersion and Risk Assessment of Bacterial Aerosols Emitted from Rotating- Brush Aerator during Summer in a Wastewater Treatment Plant of Xi’an, China	WWTP with oxidation ditch process	Bacteria	Air samples: Active method—Impaction (Andersen six-stage cascade impactor, flow rate = 28.3 L/min)	Seasonal (summer)	Directly Downwind Sites: 2 m downwind 5 m downwind 10 m downwind 30 m downwind 50 m downwind 100 m downwind Lateral Sites: G1 (5 m laterally from the aerator) G2 (5 m laterally from the aerator)	Culture-based methods	Higher airborne bacteria concentrations were observed closer to the aerator.	[5]
Airborne bacteria in a wastewater treatment plant: Emission characterization, source analysis and health risk assessment	WWTP with anaerobic–anoxic–oxic process	Bacteria	Air samples: Active method—Impaction (Quartz membranes (90 mm, Whatman QM-A), flow-rate = 100 L/min and TH-150)	Seasonal (spring, summer and winter)	The WWTP has various treatment stages, including CS (possibly activated sludge), AGC (grit chamber), PST (primary settling tank), AnT (possibly anoxic tank), AeT (aeration tank), and SST (secondary settling tank). Indoor facilities like CS and SDH (sludge dewatering with decanter centrifuges) were compared with outdoor facilities like AGC, PST, and AeT.	High-throughput sequencing techniques	Concentrations varied by site and season. Treatment stages were significant emission sources.	[27]
Quantifying the Relationship between SARS-CoV-2 Wastewater Concentrations and Building-Level COVID-19 Prevalence at an Isolation Residence: A Passive Sampling Approach	no data	SARS-CoV-2	Passive method (tampons made from rayon with a polyester string)	Seasonal (spring)	Approximately 190 feet from the isolation residence, and the wastewater influent at this location was restricted to the isolation building	RT-qPCR	The virus was detected over 16 weeks, demonstrating its feasibility for identifying residential halls with infected individuals. The daily viral wastewater load showed a positive association with the building’s COVID-19 prevalence.	[35]
Assessment of airborne virus contamination in wastewater treatment plants	no data	Adenovirus (AdV); Norovirus (NoV); Hepatitis E Virus (HEV)	Air samples: Active method—Impaction (3 μm pore size, 25 mm gelatine filters embedded in standard cassettes using MSA Escort Elf or SKC pocket pump 210–1002, flow rate = 4 L/min)	Seasonal (summer and winter)	Inside (Enclosed Area): One sample was collected in the enclosed area, specifically near the water inlet. The sampling point inside was close to the rake that removes large particles from incoming water. Outside (Unenclosed Area): Another sample was collected in the unenclosed area, specifically above the bubbling aeration basin; 123 air samples from 31 WWTPs.	qPCR	AdV-F was present in all WWTPs during summer and 97% during winter. Concentrations were higher in summer, reaching a maximum of 2.27 × 10^6^ genome equivalent/m^3^. AdV-E/D were detected in winter, only in a few samples. NoV was detected in only 3 out of 123 air samples, with concentrations below quantification limits. HEV was not detected in any of the samples.	[20]
Airborne bacteria and fungi in a wastewater treatment plant: type and characterization of bio-aerosols, emission characterization and mapping	no data	Bacteria and Fungi	Air samples: Active method—Impaction (One-Stage Andersen cascade impactor, flow rate = 28.3 L/min)	Seasonal (spring, summer and winter)	ETP (Entrance of Treatment Plant), Gch (Grit Chamber), SDB (Sludge Drying Bed), Aea tank (Aeration tank), and Lab (Laboratory Building). Within the mentioned areas, specific points were chosen for sampling, such as the pumping station, additional points in SDB, Gch, Aea tank, and the laboratory.	PCR; biochemical tests: urease, oxidative fermentative (OF), oxidase, catalase, triple sugar iron (TSI), eosin methylene blue (EMB), and Indole-Methyl red-Voges-Proskauer-Citrate (IMViC) test	Various bacteria were identified (some with pathogenic potential), and fungi were present in the air of the WWTP. Bacterial concentrations exceeded the standards, as is the case of *Staphylococcus* and *Enterobacteriaceae*. Fungal concentrations varied seasonally and by location. The relationship between meteorological parameters and bio-aerosols was explored, with temperature showing significance. Particulate matter, especially PM10, correlated significantly with fungal concentrations.	[33]
Exposure to Bioaerosol from Sewage Systems	no data	Mesophilic Bacteria; Coliform Bacteria; *Aspergillus fumigatus*	Air samples: Active methods—Impaction (MAS-100, flow rate = 100 L/min) and Impinger (SKC Biosampler, flow rate = 12.5 ± 0.1 L/min)	Seasonal (summer and winter)	At hospital sewage (K1), relief chamber of a combined sewage overflow (K2) and in the area of a city treatment plant (K3); 30 air samples	Culture-based methods (Blood agar was used for mesophilic bacteria and *Aspergillus fumigatus*, Endoagar for coliform bacteria, Coli-ID agar for *Escheriachia coli*, Hektoenagar for *Salmonella* sp., and *Camplylobacter* agar with selective supplement for *Camplylobacter* sp.)	Mesophilic Bacteria Concentrations: Location K1 had concentrations ten times higher than ambient air, attributed to the small chamber size. Location K2 exhibited concentrations comparable to ambient air, possibly due to the large size and good ventilation of the relief chamber. In the encased grit chamber (K3), mesophilic bacteria concentrations were significantly higher than in K1, K2, and ambient air. Coliform bacteria concentrations were generally low, with the highest load found in the encased grit chamber (K3). Coliform bacteria were infrequently found in aerosols of wastewater plants. *Aspergillus fumigatus* was detected at all sampling sites both indoors and outdoors.	[21]
Methicillin-Resistant *Staphylococcus aureus* (MRSA) Detected at Four U.S. Wastewater Treatment Plants	WWTP with primary, secondary and tertiary treatment processes	Methicillin-resistant *Staphylococcus aureus* (MRSA)	Material collection (Grab Samples—Samples were collected in 1-L sterile polyethylene Nalgene^®^ Wide Mouth Environmental Sample Bottles)	1 year longitudinal study	Mid-Atlantic WWTP1 Mid-Atlantic WWTP2 Midwest WWTP1 Midwest WWTP2; 44 grab samples were collected	Gram stain; coagulase and catalase tests; PCR	MRSA was detected in 50% of wastewater samples, at all WWTPs studied. MSSA (Methicillin-Susceptible *Staphylococcus aureus*) was also detected in 55% of wastewater samples, at all WWTPs. The occurrence of MRSA and MSSA varied across WWTPs, sampling dates, and sampling locations. MRSA isolates showed resistance to multiple antibiotics, including those approved for treating MRSA infections. MSSA isolates also exhibited antibiotic resistance patterns that varied by WWTP. In total, 93% of MRSA isolates were multidrug-resistant (MDR), while 29% of MSSA isolates were MDR.	[34]
Characterization and source analysis of indoor/outdoor culturable airborne bacteria in a municipal wastewater treatment plant	Municipal WWTP with anaerobic–anoxic–oxic treatment process	Airborne Bacteria, Enterobacteriaceae and Opportunistic Pathogens	Air Sample: Active method—Impaction (Andersen six-stage cascade impactor, flow rate = 28.3 L/min)	Seasonal (spring, summer, autumn and winter)	Four specific sampling sites were selected within the plant: fine screens room (FS), aeration tank (AT), sludge dewatering house (SDH), and an external upwind control site; 48 air samples	Culture-based methods; Illumina MiSeq sequencing	FS had over ten times higher concentrations of culturable airborne bacteria compared to the outdoor aeration tank. Particle size distribution of culturable airborne bacteria varied between sampling sites. Enterobacteriaceae and opportunistic pathogens were detected indoors, primarily sourced from wastewater and sludge (were not detected outdoors).	[28]
Assessment of indoor airborne contamination in a wastewater treatment plant	Municipal WWTP with preliminary, primary, secondary, tertiary and sludge treatments, and deodorization processes	Bacteria and Fungi	Air Sample: Active method—Impaction (MAS 100, flow rate = 100 L/min)	Seasonal (summer, autumn and winter)	Bar Rack Chamber SEDIPAC 3D (Degritting/Degreasing/Primary Sedimentation Facility) Secondary Sedimentation Tanks (Two Locations) Sludge Thickener Sludge Dehydration Chamber Sludge Disposal Area Outdoor Control Sampling Point	Culture-based methods (TSA for total bacteria, Mannitol salt agar and MAC for Gram-positive and Gram-negative bacteria, respectively, and DG18 for total fungi)	Out of 3 sampling campaigns, in the first one (with the highest ambient temperature) the total airborne bacteria and fungi concentrations were the highest. Gram-positive bacteria were the most dominant, and *Aspergillus*, *Penicillium*, *Cladosporium*, and *Alternaria* were the most common fungi.	[15]
Estimation of health risks caused by exposure to enteroviruses from agricultural application of wastewater effluents	WWTPs with conventional activated sludge processes	Fecal Coliforms and Enteric Viruses	Material collection (effluent samples were collected in 1-L sterile glasses)	Seasonal (spring, summer, autumn and winter)	30 effluent samples (15 from each WWTP)	Culture-based methods	A high fecal coliform concentration was observed in the WWTPs. Enteric viruses were also detected, peaking in summer/autumn. There was a high risk for farmers (EV infection and disease burden) and risk for lettuce consumers, exceeding WHO guidelines.	[32]

In terms of sampling strategy, seven papers opted to conduct sampling in two seasons (25%) [16,17,20,21,23,24,36]. Four studies (14.29%) were carried out in a single season [5,13,22,35] while another four studies covered all four seasons (14.29%) [4,26,28,32]. Furthermore, three authors conducted sampling activities across three seasons (10.71%) [15,27,33]. Five studies (17.86%) focused on a one-year longitudinal study [14,19,30,31,34]. Additionally, three studies (10.71%) differentiated sampling procedures between warm and cold seasons [6,29,37], whereas two studies did not specify the timing of their sampling activities (7.14%).

Air sampling emerged as the most employed technique, utilized in 24 out of 28 studies (85.71%) [4,5,6,13,14,15,16,17,18,19,20,21,22,23,24,25,27,28,29,30,31,33,36,37]. Active air sampling was carried out in 22 papers (78.57%) [4,5,13,14,15,16,17,18,19,20,21,22,23,24,25,27,28,30,31,33,36,37], and among these, the impaction method was predominant, with 19 studies (67.86%) [4,5,13,14,15,16,17,19,20,21,22,23,24,25,27,28,30,33,37] using different sampling devices such as the six-stage (32.14%) [5,16,19,22,23,24,25,28,37] and single-stage impaction (25%) [13,14,15,17,21,30,33]. The impingement method was employed in seven studies (25%) [4,13,14,21,25,31,36], while only five studies (17.86%) [4,13,14,21,25] utilized both impaction and impingement methods, simultaneously. Four studies used the filtration method (14.29%) [17,18,19,37]. Regarding passive sampling, it was employed in five studies (14.29%) [6,13,14,29,35], the 1/1/1 standard was used in two studies (in accordance with the microbiological sampling index of the air, a plate is placed at 1 m height, at 1 m distance to the (possible) source of contamination, and it is performed for a period of 1 h) (7.14%) [6,29], and surface samples were used in two papers (7.14%) [13,14]. Active and passive sampling strategies were carried out simultaneously in 2 out of the 28 studies (7.14%) [13,14]. Regarding the type of microbial contamination assessed, the majority of the studies (50%) [5,6,16,17,19,22,23,24,25,27,28,34,36,37] focused only on bacteria, while three studies (10.71%) [13,14,29] focused solely on fungi, and another three (10.71%) [20,31,35] evaluated only virus exposure. Six studies (21.43%) [4,15,18,21,30,33] included both fungi and bacteria, while one (3.57%) [18] examined bacteria, fungi, and viruses collectively, and another (3.57%) [32] assessed bacteria and viruses together.

Culture-based methods were the most frequently employed assays, utilized in 20 out of 28 studies (71.43%) [4,5,6,13,14,15,16,17,18,21,22,23,24,25,26,28,29,30,32,37]. Among the most used culture media, for fungal growth, three studies used MEA (Malt Extract Agar) (10.71%) [4,13,14], two studies Saboraud dextrose agar (SDA) (7.14%) [29,30], and one used Dichloran Glycerol agar (DG18) (3.57%) [18]. For bacteria, four studies used Tryptic Soy Agar (TSA) (14.19%) [4,15,30,37], three studies MacConkey Agar Medium (MAC) (10.71%) [15,22,24], and only one used Mannitol Egg Yolk (MYP) (3.57%) [24]. Nine of these studies only used one culture media for bacteria and/or fungi growth [4,6,13,14,16,17,23,29,30], and four used more than one culture media for bacteria growth [15,21,24,37]. In total, five studies did not mention the culture media used (17.86%) [5,25,26,28,32]. 

Molecular techniques were applied in 19 papers (67.86%): 13 employed Polymerase Chain Reaction (PCR) (46.43%) [4,6,13,14,20,23,25,26,31,33,34,35,37], and 6 used sequencing (21.43%) [22,25,26,27,28,36]. In PCR assays, to target bacterial strains, 5 out of 28 studies amplified bacterial 16S rRNA using universal primers (17.86%) [6,22,23,25,33], one amplified *Eschecrichia coli* MG1655 (3.57%) [36], and another used Chis150f and Clostlr primers for *Clostridium* sp. (3.57%) [16]. To detect bacterial pathogenic species, for *Staphylococcus aureus*, the primers used were NUC1 and NUC2 to target the NUC gene (3.57%) [34]. Another study targeted bacterial populations from human flora, such as *Escherichia coli*, *Klebsiella pneumoniae*, and *Pseudomonas aeruginosa*, and bacterial populations from freshwater environments such as *Aeromonas hydrophila* (3.57%) [37]. Three out of twenty-eight studies focused on antibiotic resistance profiling, one for MRSA using ECA1 and MECA2 primers for the amplification of mecA gene (3.57%) [34], one study (3.57%) used PCR coupled with gel electrophoresis to detect antibiotic resistance genes, such as sul1, sul2, sul3 for sulfonamide, tetA, tetC, tetO, tet W for tetracycline and integrons (intl1, intl2 and intl3) [4], and other amplified blaNDM, blaKPC, blaOXA-48, blaIMP, and blaVIM genes for Carbapenem-Resistant Citrobacter spp. (CRCS) (3.57%) [26]. For targeting viruses, two papers focused on SARS-CoV-2 (7.14%), one on the N1 and N2 unique genes [35] and one on RdRp, ORF-1ab, and N [31]. In one study (3.57%), three duplex qPCR were performed to target NoV180 GGII/RYMV and HEV/RYMV for RNA viruses, and AdV-40/AdV-E/D for DNA viruses [20]. For fungi, PCR was used to target *Aspergillus* sections such as *Flavi* (toxigenic strains), *Fumigati* and *Circumdati* in one paper [14], and only *Aspergillus* section *Flavi* in another [13]. Regarding sequencing methodologies, three out of six studies targeted the identification of airborne bacteria [22,27,28]; one targeted 16 rRNA to delineate the composition and similarities of microbiomes in water and air samples [36], one targeted taxonomic species of CRS [26], and one used sequencing to evaluate the positive clones of *Eschecrichia coli* [25]. In total, 11 out of 28 studies (39.29%), applied both molecular techniques and culture-based methods [4,6,13,14,16,22,23,25,26,28,37].

Among the species identified, the most prevalent Gram-positive bacteria were *Staphylococcus* sp. (21.43%) [17,19,24,30,33,34], *Bacillus* sp. (7.14%) [6,23] and *Clostridium* sp. (3.57%) [16], and the most prevalent Gram-negative were *Escherichia* sp. (7.14%) [22,24] and *Legionella* sp. (3.57%) [36]. *Aspergillus* sp. (17.86%) [13,14,15,21,30], *Cladosporium* sp. (10.71%) [15,29,30] and *Alternaria* sp. (10.71%) [15,29,30] dominated the fungal presence. One study focused on the dissemination of Methicillin-resistant *Staphylococcus aureus* (MRSA) [34], while another investigated the occupational exposure to *Staphylococcus aureus* in wastewater treatment plants, particularly focusing on antibiotic resistance [17].

## 4. Discussion 

WWTPs are crucial for the implementation of the zero-waste strategy which is in the scope of the EC’s circular economy management. Interestingly, the geographical distribution of the analyzed studies corroborated the urge for tackling WWTPs’ pollution threat and to answer to the determined environmental goals worldwide. In agreement with previous reviews held in different settings, such as poultries [9] and sawmills [10], no standardization was observed in the sampling campaigns performed, as well as in the assays employed. Furthermore, the lack of standardized contextual information retrieved through the developed studies hinders the possibility to identify the environmental variables that contribute effectively to the occupational exposure assessment, as well as to propose suitable recommendations to avoid microbial exposure and dissemination [38]. In fact, the contextual information (e.g., implemented occupational health measures, training on safety issues related to the working tasks, cleaning practices, ventilation conditions, number of workers in each workstation, protection devices used by workers), when retrieved, should allow the identification of the most critical scenario and, thus, the selection of proper sampling sites following the “worst case scenario” approach as a first step for exposure assessment. In those sampling sites considered as the most critical, besides the environmental sampling campaign, nasopharyngeal swabs should be collected from the workers’ nose to obtain additional information regarding workers’ exposure. In previous studies, nasopharyngeal swabs were also taken to assess MRSA prevalence in workers from different occupational settings [39] or to corroborate the predominant fungi present in the Portuguese cork industry and, more specifically, exposure to *Penicillium* section *Aspergilloides* [40]. In addition, this approach can help occupational health services to prioritize multiple interventions in workers’ education or even in personal protection device (e.g., gloves, respiratory protection devices) selection and replacement frequency.

The assessment of microbial dynamics in WWTPs is critical for ensuring public health and environmental safety. Seasonal evaluation plays a crucial role in this assessment, particularly given the influence of global warming and human activities, such as intensive agriculture, on microbial ecology [41,42]. In fact, recent studies [43,44] suggest that these factors contribute to the emergence of new fungal species, underscoring the need for comprehensive monitoring strategies. Recognizing the prevalence of research in specific regions and climatic periods is vital for contextualizing findings and understanding their implications for human health. Moreover, linking environmental exposure to health outcomes emphasizes the importance of establishing regulatory limits based on health considerations. This underscores the interconnectedness of the environment, exposure, and health outcomes, necessitating comprehensive regulatory frameworks.

Most of the selected papers (78.57%) exclusively applied active sampling methods, with impaction being the most frequently used method (67.86%). This sampling strategy is based on culture-based methods, which only allows the evaluation of culturable microorganisms, and thus microorganisms’ cells that are potentially damaged due to the high velocity of the airflow are not isolated [10,37,45]. Furthermore, it is critical to emphasize that air is not uniform in place or time and that it is always subject to change based on the kind and intensity of the activities occurring there or other environmental variables (e.g., climate conditions) [36,46]. Thus, the sampling period must match the setting of the research and the work being developed in that specific environment. Passive sampling methods were applied in only a few of the analyzed studies as a stand-alone method (14.29%). However, passive sampling methods are expected to be more reliable than active sampling methods since they can collect contamination over longer periods, allowing to cover all the changes that may happen in the environment [47] such as the ventilation, environmental features [48], water infiltrations and damage [49], as well as the type of task being developed in that workplace [10,50,51]. Additionally, passive sampling methods allow the combination of different assays such as culture-based methods and molecular tools increasing the accuracy of obtained results [52]. Although only two papers (7.14%) used active and passive sampling methods together, this should be the trend to follow, since this allows each sampling methods’ drawbacks to be overcome [10].

The fact that culture-based methods are primarily used for microbial characterization as standard methods for microbial assessment [53,54] might justify its frequent use among the selected papers (71.43%). This methodology is crucial to estimate health risks, since microorganisms’ viability can limit microorganisms’ inflammatory and/or cytotoxic potential [10,54,55]. Despite the advantages, conventional approaches may underestimate results since incubation temperatures and culture conditions may favor specific species. Plus, typical procedures may not always be effective in cultivating certain common microorganisms [53]. Furthermore, a recent study [53] highlights the importance of culture media selection and its significant impact on fungal counts and species diversity. Although some studies (17.86%) did not mention what culture media were employed, accurate culture media selection is critical for exposure assessment in different environments, particularly when targeting *Aspergillus* sp. [53]. Overall, three cultural media were employed for fungal assessment (MEA, DG18, and SDA). MEA and SDA are the most used non-selective media for fungi and yeasts, whereas DG18 is a fast-growing fungi inhibitor, allowing more diversity in the growth of fungal strains [56]. MEA and DG18 have both been used alongside and have proven to be useful in the growth of *Aspergillus* species according to the matrix, sampling method employed, and indoor environment assessed [57]. For bacterial assessment, TSA was the most non-selective media related to the growth of fastidious bacteria, while MAC was the most used selective and differential media related to the growth of Gram-negative bacteria, useful for the identification of enteric bacteria [58]. MYP allows the identification of Gram-positive bacteria as *Bacillus cereus* [59]. The use of multiple culture media is fundamental for the isolation and identification of a wider spectrum of microorganisms. Also, the integration of multiple culture media and different incubation temperatures in culturomics methods (such as MALDI-TOF) permits a more precise identification of unknown isolates [60,61]. This approach allows accurate microbial characterization, particularly the rapid identification of potential pathogens. In fact, culturomics methods bridge the gap between culture-based methods and molecular techniques, providing a comprehensive assessment of bioaerosols [38].

Recently, culture-independent techniques such as PCR and genome sequencing have been demonstrated to be useful for various bioaerosol measurements [52]. Indeed, PCR and sequencing were frequently performed by the authors in the selected papers. These techniques enable the detection of non-viable microorganisms as well as their potentially allergenic components [52,62], providing more information regarding microbial diversity in the evaluated environment [9]. Molecular techniques along with culture-based methods were applied by some papers (39.29%). This strategy is highly supported, since both viable and non-viable microorganisms are considered, providing a wider microbial characterization [9,10,52], and a more accurate characterization of the exposure scenario [14]. 

Furthermore, molecular techniques development has also enabled the assessment of Antibiotic Multidrug Resistance (AMD), including resistance genes associated with bacteria contamination. Recently, the World Health Organization (WHO) released an updated Bacterial Priority Pathogens List (BPPL) 2024, in which 15 families of antibiotic-resistant bacteria were grouped into critical, high and medium categories in order to allow an effective prioritization [63]. Additionally, the European Food Safety Authority (EFSA) panel on Biological Hazards recently emitted a Scientific Opinion in which the highest priority antimicrobial-resistant bacteria (ARB) and antibiotic resistance genes (ARG) were identified in different sources, including water. Among the most relevant ARB, the panel indicated carbapenem or extended-spectrum cephalosporin and/or fluoroquinolone-resistant *Enterobacterales*, fluoroquinolone-resistant *Campylobacter* sp., Methicillin-resistant *Staphylococcus aureus* and glycopeptide-resistant *Enterococcus faecium* and *E. faecalis*. Regarding the highest priority ARGs, the panel reported *blaCTX-M*, *blaVIM*, *blaNDM*, *blaOXA-48-like*, *blaOXA-23*, *mcr*, *armA*, *vanA*, *cfr* and *optrA* genes. The EFSA report also evidenced the existence of several data gaps regarding sources and the relevance of transmission routes and diversity of ARB and ARGs [64]. The data analyzed in this review demonstrate that antibiotic resistance profiling, including MRSA, *mecA gene* [31], sulfonamide, *sul1*, *sul2*, *sul3*, tetracycline, *tetA*, *tetC*, *tetO*, *tet W*, integrons, *intl1*, *intl2 and intl3* [4], and Carbapenem-Resistant *blaNDM*, *blaKPC*, *blaOXA-48*, *blaIMP*, and *blaVIM* genes [26] is already a reality. Moreover, despite the fact that the quantitative microbial risk assessment (QMRA) of WWTPs has been classically focused on risk-based monitoring targets, it is accepted that the expansion of QMRA methodologies, to include ARG, may be key for the assessment of the relative risk of these contaminants [65]. The assessment of ARG units is crucial for the identification of relevant/high-priority sources and natural reservoirs of AMR, allowing the establishment of effective mitigation strategies in a One Health approach. Despite the fact that microbial assessment in water samples and sewage treatment plants has been carried out, the development of official monitoring strategies and effective risk assessment in sewage treatment plants is crucial. In agreement with the newly updated WHO-BPPL, which demonstrates the highly dynamic nature of AMR, increasing evidence and expert reports clearly highlight the urge to promote a comprehensive public health approach and international coordination to engage innovation and mitigation strategies [63].

On the other hand, it is important to note that ARGs identification may be influenced by the different methods employed and divergences in the measuring process from sampling to wet-lab differences, among others [66]. In addition to the multi-criteria decision analysis (MCDA) method developed by the WHO in the 2017 WHO BPPL, which is still currently applied in the 2024 WHO BPPL [63] and EFSA Panel on Biological Hazards (BIOHAZ) risk assessment monitoring (https://www.efsa.europa.eu/en/topics/topic/biological-hazards), other international multi-disciplinary networks, such as NEREUS COST Action ES1403 [67], created to access the current challenges related to wastewater reuse and high-priority concerns regarding public health and environmental protection, concluded that scientific research and environmental management should follow systematic, quantitative, and comparable ARG datasets, and reported that the research community should adopt “ARG copy per cell” [66]. Thus, the development of effective mitigation measures including new monitoring technologies, such as on-line sensors that are able to detect and quantify bacterial pathogens, ARB and ARG, is crucial, as is the implementation and improvement of links between research and policy [65]. 

The identification of the most suitable fungal indicators in WWTPs is also critical for assessing treatment efficacy, environmental impacts, and public and occupational health risks [68]. Commonly used fungal species such as *Aspergillus* sp. and *Penicillium* sp. serve as markers for organic matter removal and microbial contamination [69]. Monitoring fungal indicators enables the identification of seasonal variations, climate influences, and anthropogenic impacts on wastewater quality, essential for tailoring treatment strategies. Additionally, their presence aids in the early detection of potential health hazards, such as opportunistic pathogens or allergenic molds, ensuring the safety of both workers and the public [70]. *Aspergillus* sp. was recurrent and also the most prevalent in the selected papers; the prevalence of this genera in waste management industries has already been recognized, highlighting the need for further research regarding occupational exposure [14]. In fact, *Aspergillus* section *Fumigati* was already suggested as an indicator of harmful fungal exposure in the waste management industry [71,72,73,74] and listed by the WHO as a critical priority, considering specific criteria such as antifungal resistance, mortality, evidence-based treatment, access to diagnostics, annual incidence and complications and sequelae [75]. However, the WHO list did not consider the toxicologic potential from fungal species, neglecting the possible occupational exposure to mycotoxins, as was already reported in different occupational environments [76].

In agreement with bacteria contamination analysis, fungal assessment should also cover the resistance profile. Indeed, antifungal drug resistance is a growing global concern in both space and time. This includes newly emerging species that are resistant to multiple antifungal drugs (like the yeast *Candida auris*), as well as novel resistant variants of previously susceptible pathogens (such as the ubiquitous mold *Aspergillus fumigatus*) [77]. Because of the selection of resistant strains triggered by the growing use of triazole drugs, azole resistance in *Aspergillus fumigatus* is currently seen as an emerging hazard to global public health [78,79]. In *Aspergillus fumigatus*, azole resistance can evolve through two different pathways. First, in the setting of chronic pulmonary aspergillosis, as in individuals with cystic fibrosis, resistant strains may be chosen during or following a long-term azole therapy [79,80]. Second, the prolonged use of azole antifungals in agriculture may be connected to azole resistance [79,81,82,83,84]. Relevantly, it is reported that several antifungals cause inherent resistance in *Fumigati* cryptic species. However, selected pressure brought on by the prolonged azole therapy of patients with chronic aspergillosis or environmental selection pressures are the reasons behind the emergence of resistance acquisition in *Aspergillus fumigatus* sensu stricto. Mutations in genes engaged in the *Aspergillus fumigatus* ergosterol pathway are frequently linked to the mechanisms of azole resistance, especially in the cyp51A gene that encodes cytochrome P450 14-lanosterol demethylase, the primary target of azole antifungals [79,85,86], highlighting the relevance of using these mutations as an indicator for fungal resistance.

Considering the above, further research should be performed to select the most suitable indicators of harmful microbial contamination for this occupational setting. The lists provided by the WHO regarding fungi [86] and bacteria [87] should be considered for this endeavor, but the resistance and toxicological potential from fungi and bacteria should not be neglected. 

## 5. Conclusions

Overall, this scope review concluded what is needed to provide robust science for the guidance of occupational exposure assessments: (a) common protocol from the field (sampling campaign) to the lab (assays to employ) when aiming to perform exposure assessment in WWTPs; (b) standardized contextual information to be retrieved, allowing a proper risk control and management; (c) the selection of the most suitable microbial targets to serve as indicators of harmful microbial exposure. Filling these gaps with further studies will allow robust science to be provided to policy makers and stakeholders.

## Figures and Tables

**Table 1:** Inclusion and exclusion criteria on article selection.

Inclusion Criteria	Exclusion Criteria
Articles published from 1 January 2010 to 8 September 2023 Articles published in English Articles summarising research results from any country Original scientific articles on the subject Articles focused to microbial occupational exposure	Articles published prior to 2010 Articles published in other language Abstracts of congress, reports, reviews/state-of-the-art articles

## Data Availability

Not applicable.

## Supplementary Materials

The following supporting information can be downloaded at: https://www.mdpi.com/article/10.3390/microorganisms12061144/s1, Table S1: PRISMA Checklist.
